# The Effect on Serum Electrolytes in Patients Undergoing Elective Craniotomy for Supratentorial Brain Tumors Using PlasmaLyte A and Normal Saline as Intravenous Replacement Fluid

**DOI:** 10.7759/cureus.42656

**Published:** 2023-07-29

**Authors:** Priyanka Shrivastava, Ravi Murmu, Saurabh Suman, Saket Verma, Ladhu Lakra, Sanjay Kumar

**Affiliations:** 1 Anaesthesiology, Rajendra Institute of Medical Sciences, Ranchi, IND; 2 Biochemistry (Trauma Centre), Rajendra Institute of Medical Sciences, Ranchi, IND; 3 Anaesthesiology and Critical Care, All India Institute of Medical Sciences Deoghar, Deoghar, IND

**Keywords:** intravenous fluid, metabolic status, neurosurgery, normal saline, plasma-lyte, supratentorial brain tumours

## Abstract

Background and aim

The type of fluid which is administered to patients is very crucial and important. In this study normal saline is compared with PlasmaLyte A in patients undergoing craniotomy for supratentorial brain tumors. Generally normal saline is used in neurosurgical patients; it is seen to be associated with hyperchloremic acidosis. A balanced crystalloid, e.g. PlasmaLyte A, maintains a better metabolic status than normal saline. This study was planned to study the metabolic effects of using PlasmaLyte A as compared with normal saline as intravenous fluids in patients undergoing supratentorial brain tumour surgeries.

Methods

This is a prospective, randomized, double-blinded study in patients undergoing craniotomy for supratentorial brain tumors. Written informed consent was taken from patients and they were divided into two groups, Group A and B of 40 patients each by computer-generated random numbers. Group A received PlasmaLyte A and Group B received normal saline intra-operatively as maintenance fluid. Heart rate, mean arterial pressure, total fluid administered, serum sodium, serum potassium, chloride, lactate, pH, serum urea, serum creatinine, osmolarity, and urine output were assessed at different time intervals in both groups. Blood urea and creatinine were assessed to see acute kidney injury.

Results

There was no difference in mean values of serum sodium, potassium, lactate, serum urea, creatinine and serum osmolarity in both groups throughout the study period. However there was a rise in serum chloride and a low pH was noted in Group B. The urine output was also similar in both groups. The metabolic status of patients receiving PlasmaLyte was better than those receiving normal saline.

Conclusion

Normal saline may cause hyperchloremic metabolic acidosis which may be avoided by using balanced crystalloids. The use of balanced crystalloids should be preferred to normal saline in neurosurgical patients to ensure a better metabolic status and good clinical outcome.

## Introduction

Intravenous fluids should be given the same consideration as other pharmacologically active agents. Ringer's lactate and normal saline are the most common fluids used in neurosurgery. The osmolarity of Ringer's lactate is 278 mosm/l which is less than plasma osmolarity and can thus result in cerebral edema [1,2]. A large volume of normal saline infusion is known to cause hyperchloremic acidosis, which can lead to acute kidney injury. Hyperchloremic acidosis causes afferent arteriolar vasoconstriction, reduced glomerular filtration rate and delayed micturition following surgery [3-6]. It may have fatal consequences because it can cause coagulation abnormality, decrease myocardial contractility, alter the immune system, and harm the renal system [7]. PlasmaLyte A is a balanced crystalloid and its composition is nearly similar to plasma. Its osmolarity is 295 mosm/l. It may aid in avoiding the issues of metabolic disturbances associated with normal saline. There is very limited research comparing normal saline with PlasmaLyte in neurosurgical patients.

## Materials and methods

This is a prospective, randomized, double-blinded trial and was conducted after getting approval from the Institutional Ethics Committee. The article is written as per consort guidelines. This trial is registered in the Clinical Trials Registry, India, CTRI/2022/05/042837.

Inclusion criteria

Patients between 18 to 60 years of age and American Society of Anesthesiologist Physical status grade lll undergoing surgery for supra tentorial brain tumor were included. 

Exclusion criteria

Patients with renal dysfunction, hemoglobin <10gm/dl, electrolyte imbalance, hypersensitivity to PlasmaLyte A, surgery involving blood loss >1.5l, surgeries of more than five hours duration, and cases requiring intra-operative vasopressors were excluded.

Method

Pre-anesthetic checkup was done one day prior to surgery. Written informed consent was taken. All the patients received injection ranitidine 150mg and injection metoclopramide 10mg as premedication. Patients were divided into two groups on the basis of computer-generated random numbers. Group A received PlasmaLyte A intra-operatively and Group B received normal saline intra-operatively as maintenance fluids. Allocation concealment was done by using sequentially numbered opaque sealed envelopes. Both the intravenous fluid bottles were completely covered with dark-coloured opaque paper. PlasmaLyte A was labelled as ‘A’ and normal saline was labelled as ‘B’. Patients were taken to the operation table and all the essential monitors were attached. The sensors for bi-spectral index were applied before pre-oxygenation. Bi-spectral index was maintained at 40 to 60 throughout surgery. Pre-oxygenation was done with 100% O2 for three minutes and patients were induced with injection fentanyl 2 mcg/kg, injection propofol 1.5-2.0 mg/kg in slow incremental bolus doses titrated to the loss of vocal responsiveness. After ascertaining proper bag and mask ventilation, injection atracurium 0.5 mg/kg was given. After ventilating for three to four minutes, the trachea was intubated with the proper size endotracheal tube. End tidal carbon dioxide was monitored throughout surgery. Right subclavian vein was cannulated using the Seldinger technique. Left radial artery was cannulated for invasive blood pressure monitoring and regular acid base gas analysis. Anesthesia was maintained with N2O, O2, isoflurane and top-up doses of atracurium. The rate of infusion of either fluid was maintained at a range of 8-10 ml/kg/hour in both groups. Analysis of arterial blood gas was done at baseline (T1), at the time of dura opening (T2), one hour after dura opening (T3), at skin closure (T4), one hour postoperatively (T5), 24 hours postoperatively (T6), and 48 hours postoperatively (T7). The parameters that were observed during these time intervals were heart rate (HR), mean arterial pressure (MAP), serum sodium, serum potassium, serum chloride, lactate, pH, serum urea, serum creatinine, osmolarity, and urine output.

Statistics 

Considering a 95% confidence interval, power of 80% and precision of 5% assuming standard deviation (σ)=18 and effect size of 10, and considering attrition of 10%, the final sample size came out to be 40 in each group [8].

Data were analysed by SPSS software version 22 (IBM Corp., Armonk, NY, USA). Continuous variables are presented as mean±standard deviation. The categorical variables are presented as absolute numbers and percentages. The comparison of normally distributed continuous variables between the groups was performed using the student’s t-test. Nominal categorical data between the groups were compared using the chi-square test or Fisher’s exact test as appropriate. P<0.05 was considered significant.

## Results

Demographic characteristics of patients

It was observed that there was no significant difference in mean age (p=0.801), mean weight (p=0.672) or gender distribution (p=0.348) between both groups (Table 1).

**Table 1:** Demographic characteristics of patients All values are expressed as Mean ± SD or number of subjects(%).

	Group A	Group B	P-value
Age(years)	40.53±12.469	39.05±12.653	0.801
Weight(kg)	67.00±13.58	65.70±12.501	0.762
Gender			
Male	24(60%)	28(70%)	.348
Female	16(40%)	12(30%)	

Comparison of heart rate and mean arterial pressure

There was no significant difference in mean heart rate or mean arterial pressure ​​at different time intervals in both groups (Table 2).

**Table 2:** Comparison of mean heart rate and mean arterial pressure at various time points between both groups

		Mean±SD		P value
		Group A	Group B	
Heart rate (beats/min)	T1	84.65±11.746	83.88±9.370	0.201
	T2	87.08±15.378	82.20±12.984	0.572
	T3	82.08±15.662	78.38±15.783	0.915
	T4	80.65±16.113	75.48±16.589	0.776
	T5	91.65±15.305	89.53±13.923	0.251
	T6	93.80±11.730	90.25±11.758	0.711
	T7	93.48±12.549	90.68±11.400	0.301
Mean arterial pressure (mm Hg)	T1	94.23±6.137	95.18±6.437	0.599
	T2	92.75±6.834	93.70±7.952	0.458
	T3	89.25±6.793	90.33±7.464	0.502
	T4	87.75±8.028	87.43±8.292	0.993
	T5	93.03±9.261	94.70±10.759	0.205
	T6	94.65±6.221	95.93±6.443	0.890
	T7	95.18±5.533	95.73±5.547	0.858

Comparison of serum sodium and potassium

There was no significant change in the mean values of serum sodium and potassium at all time intervals in both groups (Table 3).

**Table 3:** Comparison of serum sodium and potassium at different time intervals in both groups

		Mean±SD		P value
		Group A	Group B	
Serum sodium (in mmol/L)	T1	139.35±3.409	138.48±2.650	0.071
	T2	139.35±3.409	139.30±2.812	0.106
	T3	139.50±3.397	140.33±2.912	0.181
	T4	139.15±3.813	141.68±3.190	0.952
	T5	139.03±2.991	142.83±3.210	0.862
	T6	139.50±2.699	142.63±2.667	0.958
	T7	139.80±2.554	142.65±2.713	0.811
Serum potassium (in mmol/L)	T1	4.10±0.307	4.00±0.304	0.941
	T2	3.95±0.450	3.90±0.304	0.423
	T3	3.88±0.516	3.85±0.366	0.218
	T4	3.75±0.494	3.60±0.496	0.133
	T5	3.68±0.526	3.35±0.483	0.673
	T6	3.88±0.516	3.83±0.385	0.322
	T7	3.97±0.460	3.90±0.304	0.806

Comparison of serum chloride, lactate and pH

The mean value of serum chloride was insignificant when compared at T1 and T2 time intervals. However there was a significant rise in mean chloride levels at T3, T4, T5, T6 and T7 time intervals (Table 4). The serum lactate showed no significant difference in both the groups throughout the study period (Table 4). There was no difference in the mean values of pH at T1, T2 and T3 time intervals. However the mean difference for change in pH was found statistically significant after the T4 time interval (Table 4).

**Table 4:** Comparison of mean values of serum chloride, lactate and pH between Group A and Group B

		Mean±SD		P value
		Group A	Group B	
Serum chlorıde (in mmol/L)	T1	100.30±2.747	100.18±2.123	0.083
	T2	100.23±2.434	101.05±2.025	0.168
	T3	100.53±2.253	102.95±1.880	0.038
	T4	100.55±2.075	104.90±1.646	0.039
	T5	100.88±2.174	106.58±1.599	0.021
	T6	101.18±2.218	108.38±1.547	0.006
	T7	101.28±2.364	109.73±1.585	0.001
Serum lactate (in mmol/L)	T1	1.05±0.221	1.08±0.267	0.361
	T2	1.18±0.385	1.13±0.365	0.771
	T3	1.85±0.580	2.05±0.504	0.122
	T4	4.10±0.591	4.05±0.639	0.823
	T5	5.45±0.986	5.33±0.859	0.357
	T6	2.55±0.749	2.58±0.747	0.932
	T7	1.78±0.530	1.80±0.620	0.630
pH	T1	7.40±0.030	7.40±.0.021	0.330
	T2	7.40±0.030	7.40±0.017	0.181
	T3	7.39±0.028	7.38±0.014	0.383
	T4	7.37±0.033	7.36±0.016	0.046
	T5	7.35±0.043	7.32±0.019	<0.001
	T6	7.38±0.039	7.34±0.016	<0.001
	T7	7.40±0.036	7.34±0.012	<0.001

Comparison of fluid administered and serum osmolarity

There was no difference in the mean values of the amount of fluid administered at T1 time (baseline) as both the groups received 500 ml of fluid respectively. Both groups received a comparable volume of intra-operative fluid at all time intervals (Table 5). The serum osmolarity of both groups showed no significant difference over the study period (Table 5).

**Table 5:** Comparison of mean volume of fluid administered and serum osmolarity in Group A and Group B

		Mean±SD		P value
Fluid administered (in ml)		Group A	Group B	
	T1	500.00±0.000	500.00±0.000	
	T2	924.75±181.631	927.50±124.092	0.113
	T3	1354.50±188.475	1361.25±171.153	0.747
	T4	1919.25±233.099	1887.50±212.359	0.633
	T5	2407.50±248.728	2315.00±247.345	0.984
	T6	4301.00±408.027	4427.50±417.555	0.581
	T7	6591.25±491.190	6647.50±584.846	0.581
Serum osmolarity (in mOsm/Kg)	T1	278.60±6.725	277.23±4.758	0.063
	T2	279.43±6.717	279.35±5.255	0.221
	T3	280.35±6.666	281.05±5.079	0.125
	T4	281.48±6.725	283.58±5.301	0.226
	T5	282.38±6.117	285.80±6.077	0.855
	T6	283.68±5.950	287.10±6.071	0.937
	T7	284.75±5.624	288.58±6.084	0.476

Comparison of serum urea and creatinine

There was no significant difference in the mean values of serum urea and creatinine at T1, T6 and T7 time intervals in both groups (Table 6).

**Table 6:** Comparison of mean values of serum urea and creatinine in Group A and Group B

		Mean±SD		P value
Urea (mg/kg)		Group A	Group B	
	T1	20.45±.3.501	20.83±.2.818	0.599
	T6	21.88±3.473	22.28±3.231	0.973
	T7	22.28±3.929	23.80±5.464	0.156
Creatinine (mg/dl)	T1	.9600±.17802	0.8375± .15638	0.068
	T6	1.0625±.16593	0.9450±.12999	0.060
	T7	1.1025±.15931	1.0425±.11959	0.061

A significant difference in urine output was observed at T6 (24 hours postoperatively) (p=0.031) time interval. Group A had a mean urine output of 1720.25±202.275 ml while Group B had a mean urine output of 1642.50±277.246 at 24 hours postoperatively (Table 7). 

**Table 7:** Comparison of urine output in Group A and Group B

		Mean±SD		P value
		Group A	Group B	
Urine output (in mL)	T1	34.50±14.088	32.75±12.555	0.876
	T2	100.63±44.016	98.63±34.492	0.247
	T3	255.75±45.285	266.00±56.013	0.419
	T4	404.50±75.683	399.00±69.533	0.332
	T5	486.00±91.028	481.50±95.771	0.778
	T6	1720.25±202.275	1642.50±277.246	0.031
	T7	2865.00±272.265	2700.00±365.850	0.124

## Discussion

The cornerstone of anesthetic management in supratentorial craniotomy is to provide optimal conditions for adequate resection of the tumour. The choice of peri-operative intravenous fluid plays a vital role in the metabolic status of the patients. The intravenous fluids with a near-normal plasma osmolarity tend to remain in the intravascular compartment reducing the likelihood of development of brain edema and raised intracranial pressure. 

The most commonly used iso-osmolar fluid in neurosurgery is 0.9% normal saline (308 mOsm/L), which is easily available and has a decent safety record as long-term fluid therapy. However administration of 0.9% normal saline in large quantities is known to produce normal anion gap hyperchloremic metabolic acidosis [9]. PlasmaLyte A is an iso-osmolar balanced salt solution (295 mOsm/L) that contains organic acid buffers like acetate and gluconate and is physiologically very similar to plasma [10].

In our study, which is a double-blinded randomized study, we compared the effects of two near-normal iso-osmolar fluids, PlasmaLyte A and 0.9% normal saline on the changes in metabolic status over a period of time. All the other possible factors affecting acid base balance such as hypoxia, hypercarbia, and MAP were continuously monitored. The patients in the study group were adequately ventilated maintaining a partial pressure of carbon dioxide (PaCO2) between 35 - 45 mm Hg. In our study, we observed that the changes in electrolytes and pH were initially comparable in both PlasmaLyte A and 0.9% normal saline group. However, with the progression of time, significant acidosis was observed in Group B. There was also a significant increase in the serum chloride levels starting from T3 time interval to the T7 time. 

A study done by Dey et al. found that the metabolic profile was better maintained with PlasmaLyte A when compared to 0.9% normal saline [11]. The brain relaxation score was well comparable in both their study groups. The authors used neutrophil gelatinase-associated lipocalin (NGAL) as a biomarker that helps in early detection of acute kidney injury as early as six hours of clinical insult. They observed a significant rise in serum NGAL levels in the normal saline group and a significant difference in the urea and creatinine levels in both the groups on the fifth postoperative day though the values were within the normal physiological range in both their study groups. In our study we used serum urea, creatinine and urine output as clinical and biochemical parameters to assess acute kidney injury. We, however, did not find any significant difference in the serum urea and creatinine levels in both groups. It may be attributable to the fact that creatinine starts to rise 24 to 72 hours after the injury has occurred [12].

A study done by Roquilly et al. on brain-injured patients found that use of balanced solutions reduces the incidence of hyperchloremic metabolic acidosis [13].

Several other non-neurological studies have been done comparing the benefits and drawbacks of normal saline and PlasmaLyte A. Shaw et al. in their study compared the effect of 0.9% normal saline and PlasmaLyte A on postoperative morbidity after open abdominal surgeries. They concluded that the use of calcium-free balanced crystalloid solution for replacement of fluid losses was associated with less postoperative morbidity compared to 0.9% normal saline [14].

Semlar et al. compared the clinical effects of a balanced crystalloid solution and 0.9% normal saline in both critically ill and non-critically ill patients. They found that the use of a balanced crystalloid solution resulted in a lower rate of the composite outcome of death from any cause such as new renal replacement therapy or persistent renal dysfunction [15]. They also did a similar study in non-critically ill patients and concluded that balanced crystalloids resulted in a lower incidence of major adverse renal events within 30 days of hospital stay [16]. 

Yunos et al. in their study compared the effects of chloride liberal vs chloride restricted intravenous fluid administration in critically ill adults. They concluded that the implementation of chloride restrictive strategy in the ICU was associated with a significant decrease in the incidence of acute kidney injury and renal replacement therapy [17].

The available literature so far suggests that balanced crystalloid solutions are better than 0.9% normal saline. Hyperchloremia has also been found to cause coagulation abnormalities which have been studied by thromboelastography and platelet aggregometry [18]. It has also been shown that it influences renal function in the postoperative period by causing renal vasoconstriction and altering tubular chloride reabsorption [19]. Acidosis following hyperchloremia is also known to increase inflammatory markers in serum [19]. A study in elderly surgical patients showed that hyperchloremic acidosis may also impair splanchnic circulation [20].

Serum osmolarity was similar in both groups A and B. Maintaining serum osmolarity is a major concern in neurosurgical patients as hypo-osmolarity causes development of brain edema and thus a rise in intracranial pressure. The mean values in serum potassium also did not show any significant difference.

The major benefit while using PlasmaLyte A as an intravenous replacement fluid is its positive effect on acid base equilibrium particularly on serum chloride levels. However, the available literature so far has very little and inconclusive evidence, as far as association between acidosis and postoperative outcome is concerned. The disadvantage of PlasmaLyte A is that it is available at a higher cost compared to normal saline. 

It was observed that the MAP, HR, and oxygen saturation (SpO2) readings were comparable in both groups. Serum chloride levels began to rise at the T3 time frame in Group B and remained significant throughout the study time frame. The pH also showed similar trends and began to decrease after the T2 time frame in Group B and remained below the normal level (7.35) throughout the end of the time frame. The rest of the biological parameters were comparable in both the study groups at all time intervals. There was a slight decrease in urine output in Group B at the T6 time frame. However, at the T7 time frame, the urine output was comparable in both the study groups. Urea and creatinine levels were comparable in both study groups at all time frames.

One of the major limitations of the present study is that we have taken creatinine to be a marker of acute kidney injury. It takes about 24 to 72 hours for creatine to rise in acute kidney injury. The study duration is 48 hours so there are chances that cases of acute kidney injury might have been missed. The most common biochemical marker for acute kidney injury is NGAL. NGAL detects acute kidney injury in as early as six hours. In the present study if NGAL had been used instead of creatinine, then early detection of acute kidney injury would have been possible [12].

## Conclusions

Normal saline 0.9% when used as a sole intravenous fluid in supratentorial brain tumour surgeries has a higher probability to cause hyperchloremic metabolic acidosis when compared to PlasmaLyte A. The metabolic parameters are better maintained with PlasmaLyte A than normal saline.

## References

[1] Tommasino C (2002). Fluids and the neurosurgical patient. Anesthesiol Clin North Am.

[2] Drummond JC, Patel PM, Cole DJ, Kelly PJ (1998). The effect of the reduction of colloid oncotic pressure, with and without reduction of osmolality, on post-traumatic cerebral edema. Anesthesiology.

[3] Li H, Sun SR, Yap JQ, Chen JH, Qian Q (2016). 0.9% saline is neither normal nor physiological. J Zhejiang Univ Sci B.

[4] McFarlane C, Lee A (1994). A comparison of Plasmalyte 148 and 0.9% saline for intra-operative fluid replacement. Anaesthesia.

[5] Chowdhury AH, Cox EF, Francis ST, Lobo DN (2012). A randomized, controlled, double-blind crossover study on the effects of 2-L infusions of 0.9% saline and plasma-lyte® 148 on renal blood flow velocity and renal cortical tissue perfusion in healthy volunteers. Ann Surg.

[6] Yunos NM, Bellomo R, Hegarty C, Story D, Ho L, Bailey M (2012). Association between a chloride-liberal vs chloride-restrictive intravenous fluid administration strategy and kidney injury in critically ill adults. JAMA.

[7] Handy JM, Soni N (2008). Physiological effects of hyperchloraemia and acidosis. Br J Anaesth.

[8] Patki AY, Padhy N, Moningi S (2018). A comparison of the effect of .9% saline versus balanced salt solution (Plasma Lyte-A) on acid base equilibrium, serum osmolarity and serum electrolytes in supratentorial neurosurgical procedures requiring craniotomy. Indian J Anesth Analg.

[9] Lima MF, Neville IS, Cavalheiro S, Bourguignon DC, Pelosi P, Malbouisson LM (2019). Balanced crystalloids versus saline for perioperative intravenous fluid administration in children undergoing neurosurgery: a randomized clinical trial. J Neurosurg Anesthesiol.

[10] Bennett VA, Cecconi M (2017). Perioperative fluid management: from physiology to improving clinical outcomes. Indian J Anaesth.

[11] Dey A, Adinarayanan S, Bidkar PU, Bangera RK, Balasubramaniyan V (2018). Comparison of normal saline and balanced crystalloid (plasmalyte) in patients undergoing elective craniotomy for supratentorial brain tumors: a randomized controlled trial. Neurol India.

[12] Haase-Fielitz A, Haase M, Devarajan P (2014). Neutrophil gelatinase-associated lipocalin as a biomarker of acute kidney injury: a critical evaluation of current status. Ann Clin Biochem.

[13] Roquilly A, Loutrel O, Cinotti R (2013). Balanced versus chloride-rich solutions for fluid resuscitation in brain-injured patients: a randomised double-blind pilot study. Crit Care.

[14] Shaw AD, Bagshaw SM, Goldstein SL, Scherer LA, Duan M, Schermer CR, Kellum JA (2012). Major complications, mortality, and resource utilization after open abdominal surgery: 0.9% saline compared to Plasma-Lyte. Ann Surg.

[15] Semler MW, Self WH, Wanderer JP (2018). Balanced crystalloids versus saline in critically ill adults. N Engl J Med.

[16] Myburgh J (2018). Patient-centered outcomes and resuscitation fluids. N Engl J Med.

[17] Yunos NM, Kim IB, Bellomo R (2011). The biochemical effects of restricting chloride-rich fluids in intensive care. Crit Care Med.

[18] Boldt J, Wolf M, Mengistu A (2007). A new plasma-adapted hydroxyethylstarch preparation: in vitro coagulation studies using thrombelastography and whole blood aggregometry. Anesth Analg.

[19] Kellum JA (2002). Fluid resuscitation and hyperchloremic acidosis in experimental sepsis: improved short-term survival and acid-base balance with Hextend compared with saline. Crit Care Med.

[20] Wilkes NJ, Woolf R, Mutch M, Mallett SV, Peachey T, Stephens R, Mythen MG (2001). The effects of balanced versus saline-based hetastarch and crystalloid solutions on acid-base and electrolyte status and gastric mucosal perfusion in elderly surgical patients. Anesth Analg.

